# Case Report: A Chinese family with MYH9-RD caused by *MYH9* p.E1841K mutation exhibiting widespread may-hegglin inclusions

**DOI:** 10.3389/fped.2025.1588675

**Published:** 2025-07-25

**Authors:** Xiaoqiang Lian, Haixia Li, Jihong Hao, Haixin Li, Ling Xu, Shuxia Zhang, Li Cao, Ruimin Li

**Affiliations:** ^1^ Department of Clinical Laboratory, Handan Central Hospital, Handan, China; ^2^ Department of Anesthesiology, Handan Central Hospital, Handan, China; ^3^ Department of Clinical Laboratory, Second Hospital of Hebei Medical University, Shijiazhuang, China

**Keywords:** inherited thrombocytopenia, *MYH9* related disease, leukocyte inclusion, giant platelets, non-muscle myosin heavy chain II-A

## Abstract

**Introduction:**

MYH9-related disease (MYH9-RD) is a rare autosomal dominant genetic syndrome characterized by congenital thrombocytopenia, with a risk of developing progressive nephropathy, sensorineural deafness, and presenile cataract. Due to its presentation of isolated thrombocytopenia, it is frequently misdiagnosed as immune thrombocytopenic purpura (ITP).

**Methods:**

A 10-year-old girl with an initial diagnosis of ITP was evaluated, based on isolated thrombocytopenia and intermittent epistaxis. Clinical assessments included peripheral blood and bone marrow smear examinations to observe cellular morphology. Family history was collected to identify potential hereditary associations. Genetic testing was performed to detect potential pathogenic mutations.

**Results:**

Peripheral blood and bone marrow smears revealed giant platelets, along with blue inclusions in neutrophils, eosinophils, and monocytes—key cytological features of MYH9-RD. Family history investigation showed thrombocytopenia in the patient's mother and maternal grandmother; additionally, the mother had mild hearing impairment, and the maternal grandmother had died of renal failure. Genetic testing confirmed the presence of the MYH9 p.E1841K mutation in the patient, which was inherited from her mother. Based on these findings, the diagnosis was revised from ITP to MYH9-RD.

**Discussion:**

This case emphasizes that MYH9-RD should be considered in the differential diagnosis of unexplained thrombocytopenia, particularly when accompanied by characteristic cytological findings (e.g., giant platelets, blue inclusions in leukocytes) and a positive family history of related manifestations. The consistency of phenotypes within the affected family supports the importance of genetic screening and long-term follow-up for relatives of confirmed cases to enable early detection and management of potential complications.

## Introduction

MYH9-related disease (MYH9-RD) represents a rare autosomal-dominant genetic disorder stemming from heterozygous mutations in the *MYH9* gene. This gene is responsible for encoding the non-muscle myosin heavy chain II-A (NMMHC-IIA) (1, 2). The cardinal clinical manifestation of MYH9-RD is macrothrombocytopenia, frequently accompanied by non-hematological symptoms, including nephritis, high-frequency hearing loss, and cataracts (3). Historically, based on diverse clinical presentations, this disorder was segmented into four distinct diseases: May-Hegglin anomaly, Sebastian syndrome, Fechtner syndrome, and Epstein syndrome (4). Around the turn of the millennium, research groups led by Kunishima et al. and Seri et al. independently identified *MYH9* mutations as the common etiological factor underlying these subtypes. This discovery led to the unification of these conditions under the umbrella term MYH9-RD (1, 2). Globally, over 300 families have reported cases of MYH9-RD, yet its incidence demonstrates marked regional variability. In Italy, the estimated incidence lies within the range of 1 in 300,000 to 1 in 400,000 [https://www.orpha.net/en/disease/detail/182050]. In Greifswald, Germany, the rate is estimated to be over 1 in 25,000 (5). In contrast, data from Asia remain relatively scarce. In recent years, with the remarkable progress of sequencing technologies, the detection rate of MYH9-RD has substantially increased. As a result, it has become one of the most frequently diagnosed hereditary thrombocytopenias (6–8). Notably, approximately 35% of MYH9-RD cases occur sporadically (9). The confluence of an elevated detection rate and a high proportion of sporadic cases has given rise to a diagnostic conundrum, greatly complicating the process of accurate identification. This study presents a case of a child with MYH9-RD harboring the p.E1841K mutation. Initially, due to isolated thrombocytopenia, the patient was misdiagnosed with immune thrombocytopenic purpura (ITP). By reporting this case, we aim to enhance awareness of the potential misdiagnoses in MYH9-RD, especially when initial symptoms are non-specific.

## Case description

**Figure 1:**
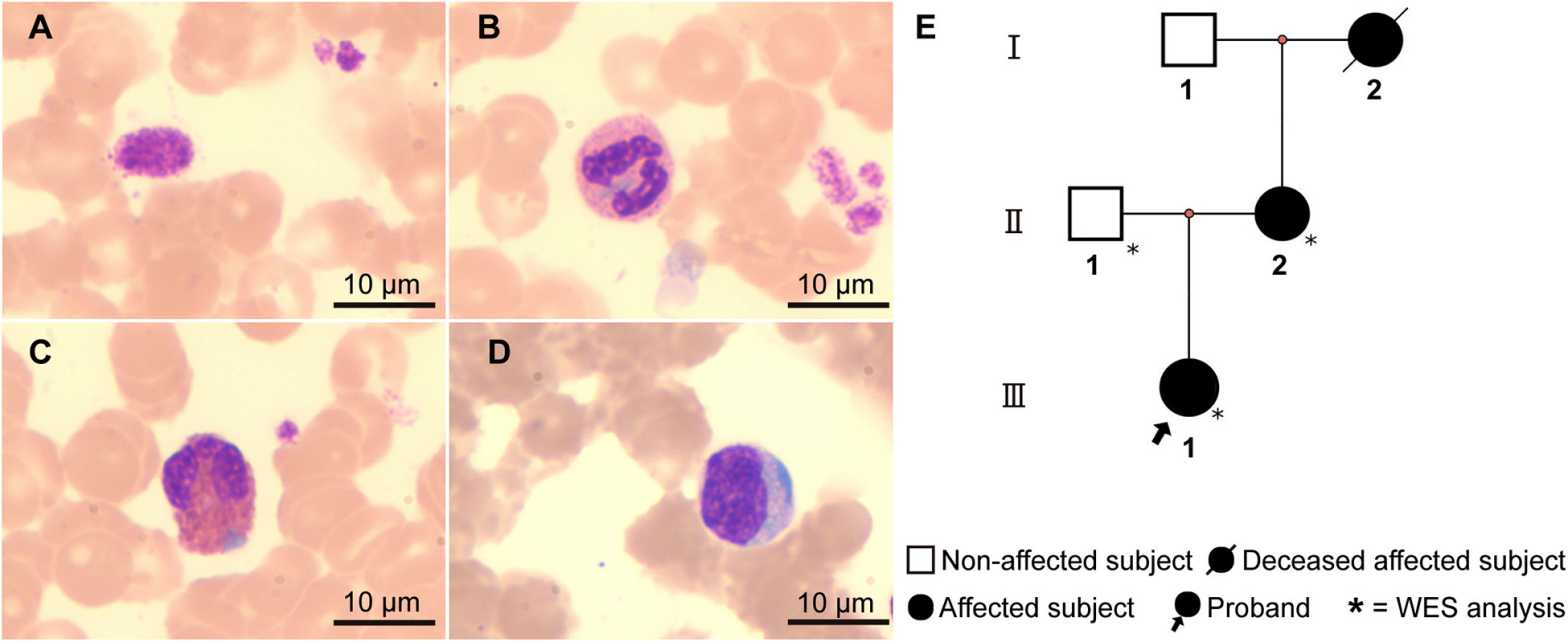
Cell morphology and the family pedigree. Peripheral blood smear of the proband (Wright-Giemsa staining, ×1,000): **(A)** Giant platelet. **(B–D)** Light blue cytoplasmic inclusions in neutrophils **(B)**, eosinophils **(C)**, and monocytes **(D)**, respectively. **(E)** Family pedigree of the proband: The proband (Ⅲ-1) presents with macrothrombocytopenia and leukocyte inclusions. The mother (Ⅱ-2) has a history of thrombocytopenia, leukocyte inclusions, and hearing impairment. The maternal grandmother (Ⅰ-2) had thrombocytopenia and died of renal failure.

The patient, a 10-year-old girl, was admitted to our hospital due to intermittent nosebleeds. Physical examination revealed no significant abnormalities. Laboratory tests showed a white blood cell count of 6.08 × 10⁹ /L, hemoglobin of 131 g/L, and platelet count of 60 × 10⁹ /L. Urinalysis and other biochemical tests showed no notable abnormalities. Initially, the clinical diagnosis was immune thrombocytopenia (ITP), and the patient was treated with intravenous immunoglobulin. However, after 5 days, a follow-up complete blood count revealed a white blood cell count of 4.51 × 10⁹ /L, hemoglobin of 129 g/L, and platelet count of 55 × 10⁹ /L, indicating no improvement from the treatment. To further investigate the cause of thrombocytopenia, the patient underwent cytomorphological examination. Notably, both bone marrow and peripheral blood smears demonstrated large platelets and blue inclusions. Some of the platelets were larger than red blood cells (Figure 1A), and the inclusions were predominantly oval or spindle, mostly located at the edges of neutrophil cytoplasm (Figure 1B). Additionally, blue inclusions were also observed in eosinophils (Figure 1C) and monocytes (Figure 1D). As reported by the proband's mother, both she and the maternal grandmother had a history of thrombocytopenia. The mother also self-reported hearing impairment, and noted that the maternal grandmother had died of renal failure. Further examination of the mother's peripheral blood smear showed large platelets and blue inclusions, while the father's peripheral blood smear was normal. Based on the clinical presentation and family history, we suspected that the patient might have the hereditary thrombocytopenia MYH9-RD.

**Figure 2:**
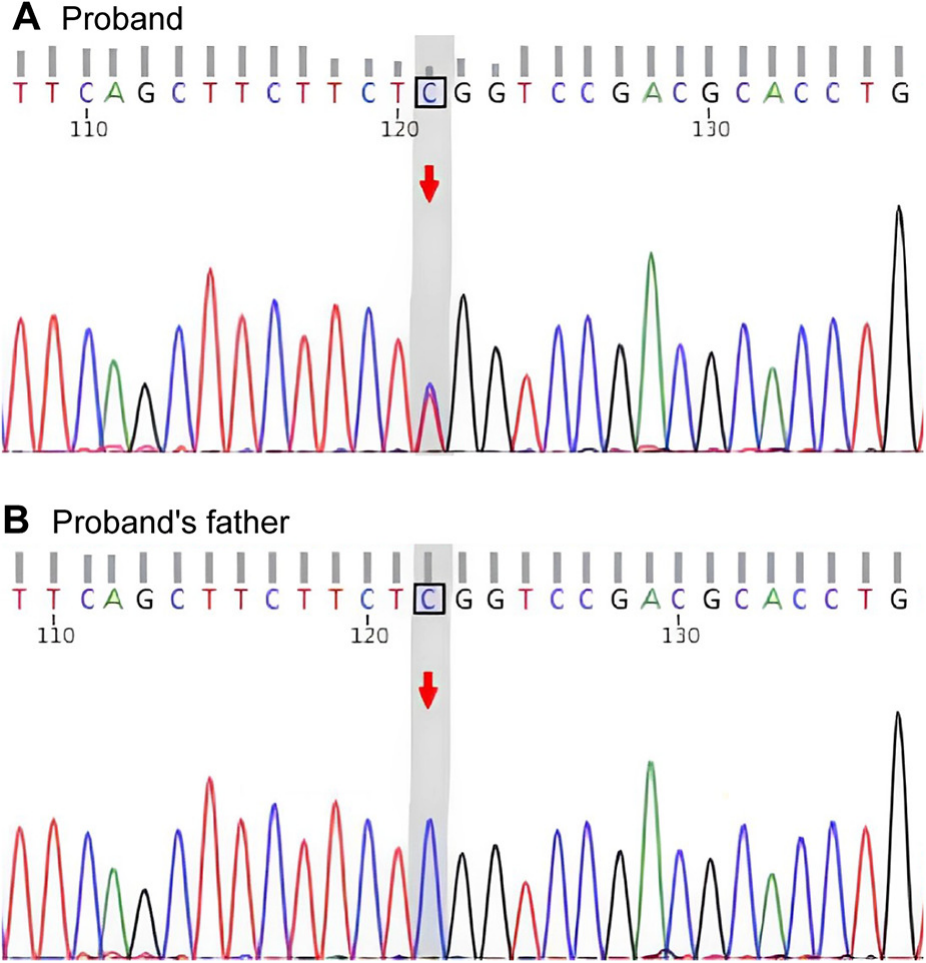
Gene sequencing. **(A)** Sequencing chromatogram of the proband showing the heterozygous *MYH9* c.5521G>A mutation, which causes the p.E1841K amino acid substitution. **(B)** Sequencing chromatogram of the proband's father showing the wild-type *MYH9* sequence.

After obtaining informed consent of this work, the whole-exome sequencing was performed on the patient and her parents. Genetic testing revealed that the patient carried the known *MYH9* c.5521G>A mutation, which leads to the substitution of glutamic acid with lysine at amino acid position 1,841 in non-muscle myosin heavy chain IIA (NMMHC-IIA) (*MYH9* p.E1841K). Segregation analysis determined that the mutation was of maternal origin and absent in the proband's father (Figure 2). Finally, a definite diagnosis of hereditary thrombocytopenia MYH9-RD was made for the child.

During hospitalization, the patient was managed for bleeding risk associated with hereditary macrothrombocytopenia using intravenous etamsylate (340 mg once daily) and vitamin C (1 g once daily) to reduce capillary permeability and enhance vascular integrity. The patient's epistaxis improved significantly with treatment, and discharge followed shortly thereafter. Upon discharge, caregivers were informed that oral maintenance therapy was not required but were advised to maintain careful daily monitoring and avoid potential bleeding triggers such as nasal trauma or vigorous physical activity. A telephone follow-up conducted one week post-discharge confirmed the absence of recurrent epistaxis or purpura. Caregivers were also instructed to continue long-term monitoring for signs of bleeding and extra-hematologic manifestations, particularly renal involvement (e.g., proteinuria) and hearing impairment.

## Discussion

The *MYH9* gene (NC_000022.11) is located on chromosome 22q12.3 and spans approximately 106,791 base pairs. It comprises 41 exons, with the first exon being non-coding. MYH9 encodes non-muscle myosin heavy chain IIA (NMMHC-IIA), a protein composed of 1,960 amino acids. Structurally, NMMHC-IIA consists of three major regions: (1) the head region, which includes a globular motor domain (encoded by exons 2–19) and a neck domain (encoded by exon 20); (2) a coiled-coil rod domain (encoded by exons 21–40); and (3) a short non-helical tail domain of 34 amino acids (encoded by exon 41) (10). The head region is responsible for actin binding and force generation through MgATPase activity, the rod domain mediates heavy chain dimerization, and the non-helical tail contains a regulatory phosphorylation site that may influence filament assembly (10, 11).

NMMHC-IIA is a critical protein extensively involved in cytodynamic processes. Mutant NMMHC-IIA abnormally aggregates within leukocytes, forming characteristic May-Hegglin inclusions, a consistent feature of MYH9-RD in clinical practice (12, 13). While severe inflammatory reactions can also lead to thrombocytopenia and the presence of blue Döhle bodies, isolated MYH9-RD typically lacks other toxic neutrophil changes. The absence of a history of infection in the proband further aids in distinguishing MYH9-RD from inflammation-induced thrombocytopenia. Moreover, the distribution of May-Hegglin inclusions under Wright-Giemsa staining has been inconsistently reported in the literature. Some studies describe these inclusions as confined to neutrophils, while others define them as present in leukocytes more broadly. Consistent with the findings of May and colleagues, our study observed that May-Hegglin inclusions are not limited to neutrophils but are also present in eosinophils and monocytes. This widespread distribution aligns with the broad expression of NMMHC-IIA across various cell types.

To date, more than 220 pathogenic variants have been identified in MYH9, the majority being missense mutations arising from single-nucleotide substitutions, while a smaller proportion consists of frameshift mutations resulting from small insertions or deletions (14). These mutations are concentrated at 21 hotspot residues, with the most frequent alterations occurring at S96 and R702 in the motor domain, and R1165, D1424, and E1841 in the rod domain—collectively accounting for approximately 80% of all reported cases (15). MYH9-RD typically presents with mild to moderate bleeding symptoms, but more significant clinical issues are often linked to Alport-like features. Studies show that approximately 30% of MYH9-RD patients are at risk for kidney damage, 16% may develop age-related cataracts, and around 60% experience hearing loss (16). Pecci et al. demonstrated a significant correlation between the MYH9-RD genotype and non-hematologic phenotypes, specifically linking mutations in the head region of NMMHC-IIA to a higher risk of kidney disease and hearing loss, while mutations in the tail region were associated with a lower risk of these conditions (3). These findings have been confirmed in subsequent studies (17, 18). In this study, the proband exhibited no significant non-hematologic features, and the mother only had mild hearing loss. While the proband's maternal grandmother died from renal failure, specific details regarding the onset and progression of her renal disease are unclear. This family's phenotypic characteristics generally align with the low-risk profile associated with the *MYH9* p.E1841K. However, in contrast, a study by Hao et al. on a Chinese family with the same mutation found no severe bleeding events in 10 patients, but 3 members died from uremia, and 4 others had varying degrees of kidney damage (19). This highlights the phenotypic heterogeneity in renal manifestations among patients with the *MYH9* p.E1841K. Although the mechanisms are not fully understood, it has been shown that the *MYH9* p.E1841K mutation alters podocyte structure, causing abnormal intracellular stress and making podocytes more susceptible to injury (20, 21). Podocytes play a critical role in renal filtration, and their dysfunction can directly impair kidney filtration, leading to renal damage. Mover, in a different low-risk family with the MYH9 p.V1516L mutation, 6 out of 11 adults developed proteinuria (22). The elevated incidence of renal damage in abovementioned families, compared to the general MYH9-RD population, suggests a phenotypic convergence within the same pedigree. Cechova, et al. found that under high-salt dietary conditions, *MYH9* p.E1841K mice exhibit significantly higher systolic blood pressure compared to the wild-type group (20). It suggesting that dietary factors may exacerbate the renal susceptibility of *MYH9* p.E1841K patients, thereby promoting the progression of renal damage. Further investigation through a within-litter feeding-controlled experiment using *MYH9* mutant mice is needed to validate this hypothesis. Therefore, considering the history of renal failure in the proband's maternal grandmother, it is advisable for the proband and other family members, who have not yet developed kidney disease, to undergo long-term monitoring of renal function. This approach is crucial for the early detection and timely management of potential kidney issues.

Chronic liver enzyme abnormalities have emerged as a new phenotype of MYH9-RD in recent years, in addition to the classic hematologic triad and Alport-like features. Pecci et al. conducted a detailed analysis of liver enzyme profiles in 75 MYH9-RD patients, revealing that 50.7% exhibited elevated levels of aspartate aminotransferase (AST) and/or alanine aminotransferase (ALT) (23). Interestingly, this liver enzyme abnormality did not appear to correlate with the patient's age or mutation site, a finding that aligns with the results from Favier et al. (23, 24). In the present study, the proband's mother did not undergo liver enzyme testing, and the proband's AST and ALT levels were within the normal range, possibly due to the absence of exposure to hepatotoxic drugs (24). Despite this, ongoing monitoring and follow-up remain crucial to assess any potential future developments.

In conclusion, this study reports a Chinese family with the *MYH9* p.E1841K mutation, characterized by a low-risk of non-hematologic manifestations. Genotype-phenotype correlation facilitates phenotypic prediction in sporadic cases or families where Alport-like features have not yet manifested. In families with established Alport-like symptoms, phenotypic homogeneity within a pedigree can guide genetic counseling and long-term management. Additionally, this study reaffirms the characteristic wide distribution of May-Hegglin inclusions across various types of white blood cells.

## Data Availability

The raw data supporting the conclusions of this article will be made available by the authors, without undue reservation.
